# Supplementary material for: Competitive Analysis of Rumen and Hindgut Microbiota Composition and Fermentation Function in Diarrheic and Non-Diarrheic Postpartum Dairy Cows
Source: Microorganisms. 2023 Dec 22;12(1):23. doi: 10.3390/microorganisms12010023 (PMC10818870; doi:10.3390/microorganisms12010023)
Supplement: Supplementary file 1 [file microorganisms-12-00023-s001.zip › microorganisms-2738429-supplementary.pdf]

Table S1. The ingredients and chemical composition of the experimental dairy cows

Items	Percentages
Ingredients, %	
Alfalfa hay	15.24
Oat hay	4.21
Whole plant corn silage	38.5
Corn grain, steam flaked	15.15
Yeast culture	1.17
Soybean meal	13.38
Cottonseed	5.32
Molasses	2.28
5% premix	3.8
Fat powder	0.95
Nutritive values	
DM (%)	53.18
Crude protein (%)	18.08
Ether extract (%)	5.04
ADF (%)	20.65
NDF (%)	31.65
Starch (%)	25.61
NE_L, Mcal/kg	1.78
Calcium (%)	0.97
Phosphorus (%)	0.47

DM = dry matter, NDF = neutral detergent fiber, ADF = acid detergent fiber.

Premix: 1 kg of premix included Ca 160 g, Cu 630 mg, P 37 g, Mn 1200 mg, I 28 mg, Zn 2700 mg, Se 21 mg, vitamin A 925,340 IU, vitamin D3 253,458 IU, vitamin E 7,689 mg, niacin 5,980 mg.

NEL: Net energy of lactation, a calculated value according to NRC (2001).

Table S2. The milk composition and apparent total tract digestibility of the different fecal score dairy cows on 9 Days in milk

Items[1]	Groups		SEM	P-value
	LFS (n=12)	HFS (n=12)		
Milk yield, kg/d	34.35	30.75	1.54	0.158
Milk composition, %				
Fat	4.84	4.22	0.32	0.339
Protein	3.29	3.48	0.06	0.129
Lactose	4.94	5.01	0.18	0.408
TS	13.07	12.70	1.37	0.534
MUN, mg/dL	15.13	15.27	0.44	0.888
SCC, 10^3 cells/mL	117.45	84.45	13.24	0.436
Digestion, %				
NDF	53.85	55.67	1.58	0.605
ADF	46.93	50.94	1.60	0.253
EE	74.32	75.55	1.48	0.707
CP	65.80	73.48	1.96	0.058
OM	71.06	75.62	2.14	0.147

[1]LFS: low fecal score, HFS: high fecal score, TS: total solid, MUN: milk urea nitrogen, SCC: somatic cell count, BHBA: β-hydroxybutyric acid, SEM: standard error of the mean.
NDF: neutral detergent fiber, ADF: acid detergent fiber, EE: ether extract, CP: crude protein, OM: organic matter.

Table S3. The different ruminal and fecal bacteria genera in the two groups based on the Wilcoxon Rank Sum Test P value

Items	Groups		SEM	P	adjP
	HFS	LFS			
**Rumen**					
Bifidobacterium	0.70	0.22	0.128	0.028	0.77
Lachnospiraceae_NK3A20_group	2.82	1.69	0.292	0.033	0.71
Prevotellaceae_UCG-004	0.24	0.37	0.031	0.030	0.73
**Fecal**					
Bifidobacterium	4.25	1.04	0.79	0.046	0.430
Breznakia	0.05	0.01	0.01	0.028	0.380
Candidatus_Stoquefichus	0.21	0.03	0.04	0.010	0.200
Cellulosilyticum	0.33	0.05	0.06	0.003	0.090
Defluviitaleaceae_UCG-011	0.25	0.05	0.03	0.000	0.073
Eubacterium_brachy_group	0.14	0.51	0.09	0.010	0.200
Eubacterium_oxidoreducens_group	0.07	0	0.02	0.033	0.380
Family_XIII_UCG-001	0	0.02	0.01	0.033	0.369
Frisingicoccus	0.18	0	0.04	0.003	0.081
Intestinibacter	0.27	0	0.05	0.003	0.090
Lachnospiraceae_FCS020_group	0.36	0.20	0.05	0.048	0.430
Lachnospiraceae_NK3A20_group	0.06	0.72	0.17	0.002	0.079
Lachnospiraceae_UCG-001	0.36	0.04	0.06	0.003	0.063
Marvinbryantia	0.49	0.17	0.07	0.035	0.380
Mogibacterium	0.01	0.10	0.03	0.039	0.390
Peptoclostridium	0	0.01	<0.01	0.013	0.220
Prevotellaceae_UCG-004	0.11	0.65	0.11	0.029	0.380
Pygmaiobacter	0.36	0.17	0.04	0.019	0.300

LFS: low fecal score, HFS: high fecal score, SEM: standard error of the mean. adjP: false discovery rate corrected P value. Wilcoxon Rank Sum Test was used to compare the difference of fecal bacteria genera relative abundance between the two groups, and P value with false discovery rate correction < 0.05 was considered significant.
